# Running Variability in Marathon—Evaluation of the Pacing Variables

**DOI:** 10.3390/medicina60020218

**Published:** 2024-01-26

**Authors:** Ivan Cuk, Srdjan Markovic, Katja Weiss, Beat Knechtle

**Affiliations:** 1 Faculty of Sport and Physical Education, University of Belgrade, 11000 Belgrade, Serbia; 2 Faculty of Physical Education and Sports Management, Singidunum University, 11000 Belgrade, Serbia; smarkovic@singidunum.ac.rs; 3 Institute of Primary Care, University of Zurich, 8006 Zurich, Switzerland; katja@weiss.co.com; 4 Medbase St. Gallen Am Vadianplatz, 9000 St. Gallen, Switzerland

**Keywords:** endurance, speed, long-distance running, evaluation, coefficient of variation

## Abstract

*Background and Objectives*: Pacing analyses for increasingly popular long-distance running disciplines have been in researchers’ spotlight for several years. In particular, assessing pacing variability in long-distance running was hardly achievable since runners must repeat long-running trials for several days. Potential solutions for these problems could be multi-stage long-distance running disciplines. Therefore, this study aimed to assess the long-distance running variability as well as the reliability, validity, and sensitivity of the variables often used for pacing analyses. *Materials and Methods*: This study collected the split times and finish times for 20 participants (17 men and three women; mean age 55.5 years ± 9.5 years) who completed the multiday marathon running race (five marathons in 5 days), held as part of the Bretzel Ultra Tri in Colmar, France, in 2021. Seven commonly used pacing variables were subsequently calculated: Coefficient of variation (CV), Change in mean speed (CS), Change in first lap speed (CSF), Absolute change in mean speed (ACS), Pace range (PR), Mid-race split (MRS), and First 32 km–10 km split (32-10). *Results*: Multi-stage marathon running showed low variability between days (Intraclass correlation coefficient (ICC) > 0.920), while only the CV, ACS, and PR variables proved to have moderate to good reliability (0.732 < ICC < 0.785). The same variables were also valid (r > 0.908), and sensitive enough to discern between runners of different performance levels (*p* < 0.05). *Conclusions*: Researchers and practitioners who aim to explore pacing in long-distance running should routinely utilize ACS, CV, and PR variables in their analyses. Other examined variables, CS, CSF, MRS, and 32-10, should be used cautiously. Future studies might try to confirm these results using different multi-stage event’s data as well as by expanding sensitivity analysis to age and gender differences.

## 1. Introduction

Optimally performed endurance activities require efficient use of available energetic resources [1]. Consequently, participants in such activities (i.e., elite and recreational athletes) must decide how and when to invest their available energy. This process is continuous, and it is known as pacing [2]. In numerous endurance sports disciplines, the optimal pacing of elite athletes is often based on the drafting possibilities and expectations/actions of their direct opponents [3,4,5]. As a result, pacing behavior adjusts towards winning a specific position in the race rather than achieving the best finish time [6]. On the other hand, recreational endurance athletes like marathoners often aim to enjoy running, avoid injuries, and (if possible) achieve the fastest race time [7]. As a result, they have more choices in selecting the best pacing strategy in accordance with their goal.

Several pacing strategies were previously reported in the scientific literature: (a) positive pacing (i.e., decreasing in speed over time), (b) negative pacing (i.e., increasing in speed over time), (c) all-out pacing (i.e., maximal possible speed from the start), (d) even pacing (i.e., constant speed over time), (e) variable pacing (i.e., significant race speed fluctuations), and (f) parabolic-shaped pacing (i.e., positive and negative pacing in different segments of the race), with no unanimous conclusion which one is the most optimal in all competitions [8]. Although, homeostasis maintenance could be a fundamental requirement for optimal performance during exercise [9]. As events take increasing time to complete (>2 min or >800 m), the optimal pacing profile appears to be more even [10,11]. Therefore, even pacing (with less speed variation) seems an obvious candidate for the optimal pacing strategy, particularly for recreational runners. It applies explicitly in prolonged locomotive events under stable external conditions (i.e., environmental and geographic), such as long-distance running, swimming, rowing, skiing, speed skating, and cycling [8]. In long-distance running, the even pacing strategy might help runners achieve a faster race time, decrease the risk of musculoskeletal injuries, and increase the pleasure of running [7,12]. In addition, with an increase in velocity, athletes use a more significant percentage of the power to overcome resistance (i.e., air or water) rather than producing forward motion [13]. Therefore, even minor speed fluctuations can result in a more significant energy cost, mainly due to acceleration [14,15]. At the same time, frequent deceleration can increase the risk of injury due to greater impact forces on the musculoskeletal apparatus [16].

Consequently, several recent studies assessed pacing in long-distance running as variability from even pacing (expressed as a percentage of speed change) [17,18,19,20]. A single dependent variable (depicting pacing variability for the entire race) was often used rather than the mean running speed for each race segment. Several reasons for such a decision can be acknowledged: (a) more complex statistical procedures can be performed to achieve robust results [18,19]; (b) pacing on two or more events held on the same track at the same time can be compared (e.g., half-marathon and marathon [18,21] or ultra-marathons of 6, 12, and 24 h [22]); and (c) the same events held in different years with a different number of checkpoints or their positions can be compared [7,17,19]. However, a potential problem with this approach is that various studies have utilized different variables to assess pacing variability in long-distance running [17,18,21,23]. At the same time, the reliability, validity, and sensitivity, as well as pacing variability, have not been evaluated in long-distance running, although pacing analysis for long-distance running disciplines has been in researchers’ spotlight for several years [18,24]. In addition, half-marathons and marathons are increasingly popular among recreational and professional runners [25]. Contrary to long-distance running, indices of pacing variability have been evaluated in other endurance sports, such as kayaking [26], swimming [27,28,29], cycling [30,31], and middle-distance running [32].

Assessing pacing variability in long-distance running is hardly achievable since runners must repeat long-running trials for several days. Furthermore, all studies mentioned above were performed in laboratory settings (e.g., treadmill running or kayak ergometer) or in time trial settings (swimming and cycling) instead of real race situations. Potential solutions for these problems could be multi-stage long-distance running disciplines. These running events are similar to Grand Tours in cycling (e.g., Tour de France, Giro d’ Italia). They include daily running a marathon for several consecutive days [33]. Multi-stage running events have a long tradition, with a recent increase in popularity [34].

Therefore, this study aims to assess the long-distance running variability as well as the reliability, validity, and sensitivity of commonly used pacing variables. For that purpose, this study used the official results of the Bretzel Ultra Tri Challenge Marathon (i.e., multi-stage marathons). We hypothesized that the results would show low pacing variability because of the athletes’ experience and stable weather throughout racing days. We also hypothesized that some pacing variables could present higher reliability, sensitivity, and validity than others, thus providing a fundamental tool for future studies interested in pacing strategies. Using potentially reliable, valid, and sensitive variables can help to obtain more robust results of the pacing analysis in long-distance running. As a result, sports scientists and coaches could help runners manage their energy better, thus avoiding burnout and injuries and achieving their running goals.

## 2. Materials and Methods

**Figure 1:**
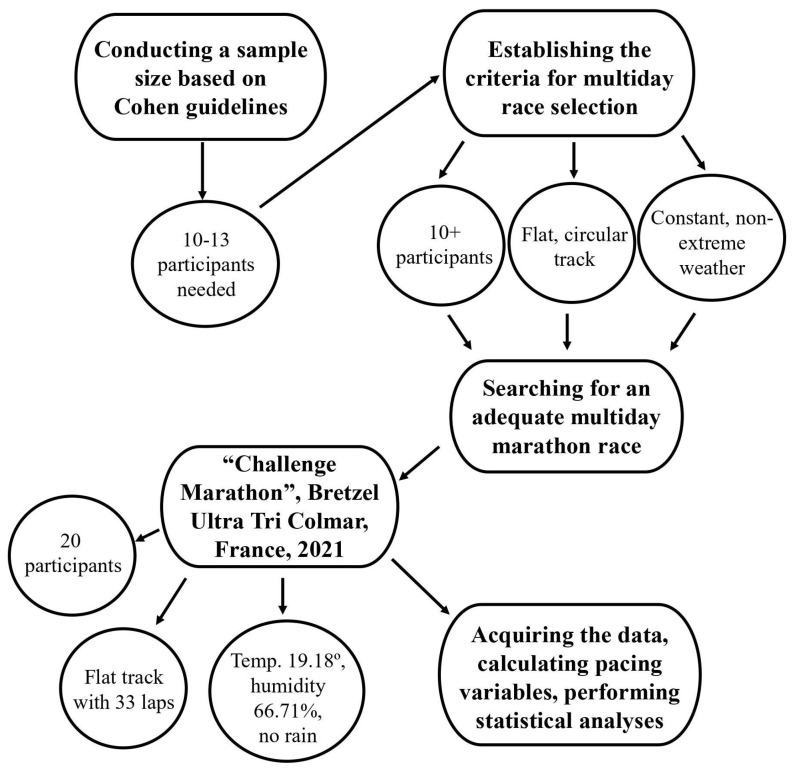
Flow chart of the study design and methodology.

The study design and methodology are graphically presented in Figure 1.

### 2.1. Ethical Approval

This study was approved by the Institutional Review Board of Kanton St. Gallen, Switzerland, with a waiver of the requirement for informed consent of the participants as the study involved the analysis of publicly available data (EKSG 01/06/2010). The study was conducted following recognized ethical standards according to the Declaration of Helsinki adopted in 1964 and revised in 2013.

### 2.2. Participants

We conducted a sample size estimate based on Cohen’s guidelines [35]. With an alpha level of 0.05, a power of 0.8, and 4 repeated measurements, 10 to 13 participants appear to be necessary for further analyses. Additionally, we also searched for previous methodological studies evaluating variability in other endurance sports disciplines. These studies included 10 to 20 participants, both men and women [26,29,30,32], and our sample size analysis confirmed that this number of participants is sufficient to test the proposed hypotheses. Therefore, this study collected each day’s split times and finish times for 20 participants (17 men and three women; mean age 55.5 years ± 9.5 years) who completed the entire race (i.e., “Challenge Marathon”). All participants` split and daily race times were obtained from the race director.

### 2.3. The Race

The multiday marathon running race was held as part of the Bretzel Ultra Tri in Colmar, France, in 2021 (https://bretzelultratri.com/challenge-marathon/). Results from four days were obtained for further analysis. The race started at 10:30 am on the first day and 10:00 am on each consecutive day. The race was held on a flat asphalt road with no elevation along a river, mainly in the forest shade on the loop track. Each lap was approximately 1279 m long; thus, 33 laps were needed to complete a marathon (42,195 m). The average 4-day temperature (hourly from 10 am to 4 pm) was 19.18° Celsius (ranging from 16 to 22°), with 66.71% humidity (ranging from 46 to 91%) and no rain.

### 2.4. Data Analysis

Individual split times and finish times for each day were initially acquired from the official race website www.bretzelultratri.com/fr/epreuves/challenge-marathon (accessed on 25 September 2021). Thirty-three split times were obtained for each marathon (approximately 1279 m per split). Each 1279 m split time was measured electronically using a shoe chip. The race weather data were obtained from https://kachelmannwetter.com/de/messwerte/haut-rhin (accessed on 10 October 2021) for Colmar in hourly intervals from 10 am to 4 pm. This time interval was selected because all runners ran all marathons in less than 6 h. The final phase of data analysis consisted of calculating different pacing variables.

#### 2.4.1. Dependent Variables

Note that all dependent variables were calculated independently for each runner:

- Mean speed (MS). The MS for each lap and the MS for each marathon race were calculated based on the time needed to complete the distance.
- Coefficient of variation (CV). CV was calculated as the standard deviation of the participant’s laps MS divided by race MS times 100 [22,36,37].
- Change in mean speed (CS). The percentage of MS change in each lap in relation to the race MS was calculated. CS was calculated as the mean of all lap percentages [20,38].
- Change in first lap speed (CSF). The percentage of MS change in each lap in relation to the first lap MS was calculated. CSF was calculated as the mean of all lap percentages [3,39].
- Absolute change in mean speed (ACS). Percentage of MS change in each lap in relation to the race MS was calculated, where all percentages were presented in absolute (i.e., positive) values. ACS was calculated as the mean of all lap percentages [18,19].
- Pace range (PR). The fastest and slowest lap MS were identified. These laps were then expressed as a percentage faster or slower than the race MS. Each individual’s fastest lap MS was named “positive range”, while the slowest lap MS was called “negative range”. The absolute sum of the positive and negative ranges was calculated to obtain PR [23,40].
- Mid-race split (MRS). MRS was calculated as a percentage of MS change in the second half of the race in relation to the MS of the first half of the race [7,41,42].
- First 32-10 split (32-10). The 32-10 split was calculated as a percentage of MS change in the last 10 km in relation to the MS of the prior 32 km of the race [17].

#### 2.4.2. Independent Variable

Three performance groups were established to test the different pacing variables’ sensitivity. Groups were visually binned based on the mean speed of all four marathons and named: Fast (mean marathon time 3:46:24 h:min:s, ranging from 3:29:57 h:min:s to 4:01:17 h:min:s), Medium (mean marathon time 4:17:49 h:min:s, ranging from 4:01:17 h:min:s to 4:37:08 h:min:s), and Slow (mean marathon time 4:59:05 h:min:s, ranging from 4:37:08 h:min:s to 5:49:55 h:min:s) [37].

### 2.5. Statistical Analysis

Descriptive statistics were calculated as mean and standard deviation before all statistical tests. Data distribution normality was confirmed by the Kolmogorov–Smirnov test and visual inspection of histograms and QQ plots.

To assess day-to-day running variability, individual linear regressions were applied on mean speed for each of the 33 laps, separately for each day, to obtain pacing profiles. A linear regressions coefficient (r) was used to present each participant’s regression. Furthermore, one-way repeated ANOVA was performed on the Z-transformed [43] coefficients mentioned above to test differences between days.

To assess the reliability of the mean speed and pacing variables obtained from the four marathons, standard error of measurement (SEM), Coefficient of variation (CV), Intraclass correlation coefficients (ICC), and repeated one-way ANOVA were performed. Since all pacing variables were expressed as percentages, data were log-transformed for the analyses and then back-transformed according to existing methods [27,44].

The Pearson correlation coefficient was performed to assess the concurrent validity of pacing variables with regard to the “golden standard” (i.e., CV) for measuring variability in sports [45].

The Pearson correlation coefficient was also used to assess the sensitivity of the pacing analytical techniques—in particular, the relationship between different pacing analytical techniques and the mean race speed. Furthermore, one-way ANOVA with LSD post-hoc test was applied to test differences in pacing (assessed by different variables) between 3 performance groups (i.e., Fast, Medium, and Slow).

Eta squared (ŋ^2^) was calculated for the ANOVAs where the effect sizes 0.01, 0.06, and above 0.14 were considered small, medium, and large, respectively [35]. All correlation coefficients were interpreted as small, r = 0.10–0.29; moderate, r = 0.30–0.49; and large, r = 0.50–1.0 [35], whereas values of ICC less than 0.5, between 0.5 and 0.75, between 0.75 and 0.9, and greater than 0.90 were indicative of poor, moderate, good, and excellent reliability, respectively [46]. The level of statistical significance was set at *p* < 0.05. All statistical tests were performed using Microsoft Office Excel 2017 (Microsoft Corporation, Redmond, WA, USA) and SPSS 26 (IBM, Armonk, NY, USA).

## 3. Results

### 3.1. Day-to-Day Running Variability

**Figure 2:**
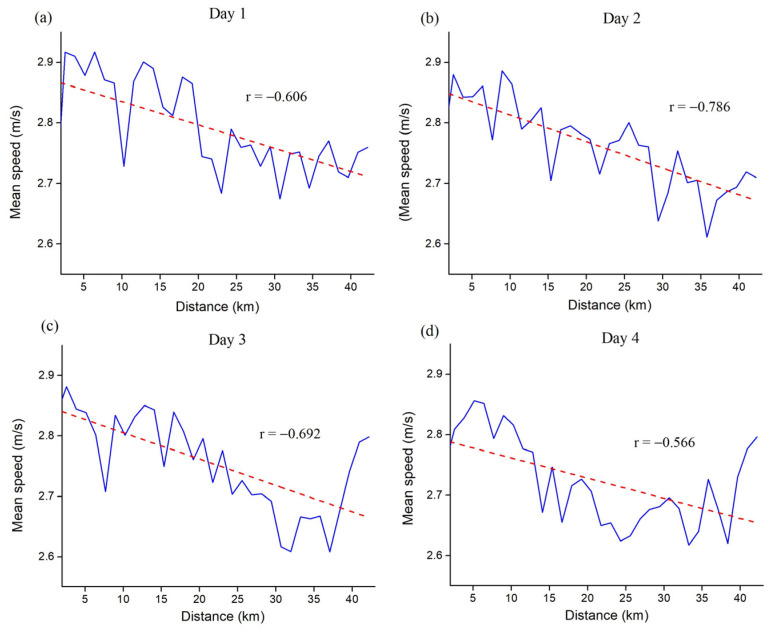
Day-to-day pacing variability presented in mean running speed (**a**–**d**). Dashed lines = mean linear regressions; r = mean linear regressions coefficient.

Day-to-day mean speed variability shows a positive pacing strategy on all four days, with a low running variability between days (Figure 2).

**Figure 3:**
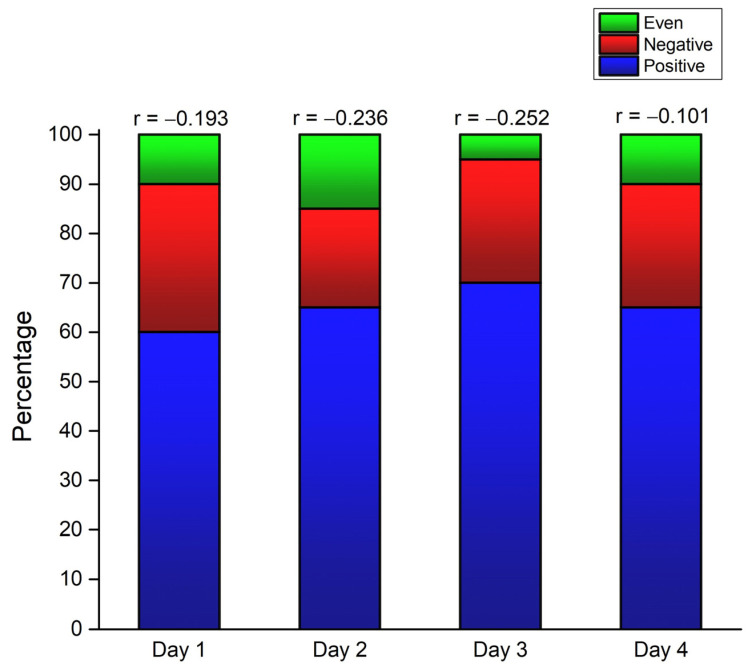
The frequency of even, negative, and positive pacing strategies adopted by 20 participants based on the individual linear regression profiles. r = mean individual linear regressions coefficient. r between −0.1 and 0.1 defines even pacing profile; r lower than −0.1 defines positive pacing profile; r higher than 0.1 defines negative pacing profile.

The frequency of participants’ positive, even, and negative pacing strategies based on the individual linear regression profiles are displayed in Figure 3. One-way repeated ANOVA performed on Fisher Z-transformed individual linear regression coefficients showed no statistical differences between the four days (F = 1.440, *p* = 0.241), thus indicating low day-to-day pacing variability.

In addition, all participants had the same pacing profile in four days (55%) or the same pacing profile in 3 out of 4 days (45%). Finally, the mean running speed showed no statistical differences between the four days (Table 1).

### 3.2. Reliability and Validity of the Pacing Variables

Mean speed showed excellent reliability indices, whereas CV and ACS showed good reliability. PR showed moderate to good reliability. Other variables proved to have poor reliability indices. Nevertheless, all variables showed no statistical significance between days.

A large and statistically significant positive correlation (r > 0.908; *p* < 0.001) was observed when ACS and PR were compared to the CV on all four days. CSF also showed a large and statistically significant positive correlation (r > 0.664; *p* < 0.001) with CV in the last three days. Other variables showed moderate-to-low and no statistically significant correlation with CV (Table 2).

### 3.3. Sensitivity of the Pacing Variables

In general, CV, ACS, and PR showed a negative and moderate-to-high correlation (r between −0.709 and −0.409) with the MS (Table 3). Correlations were also statistically significant on all variables on all days (*p* < 0.05), except for the ACS variable on day 1 (*p* = 0.07).

**Figure 4:**
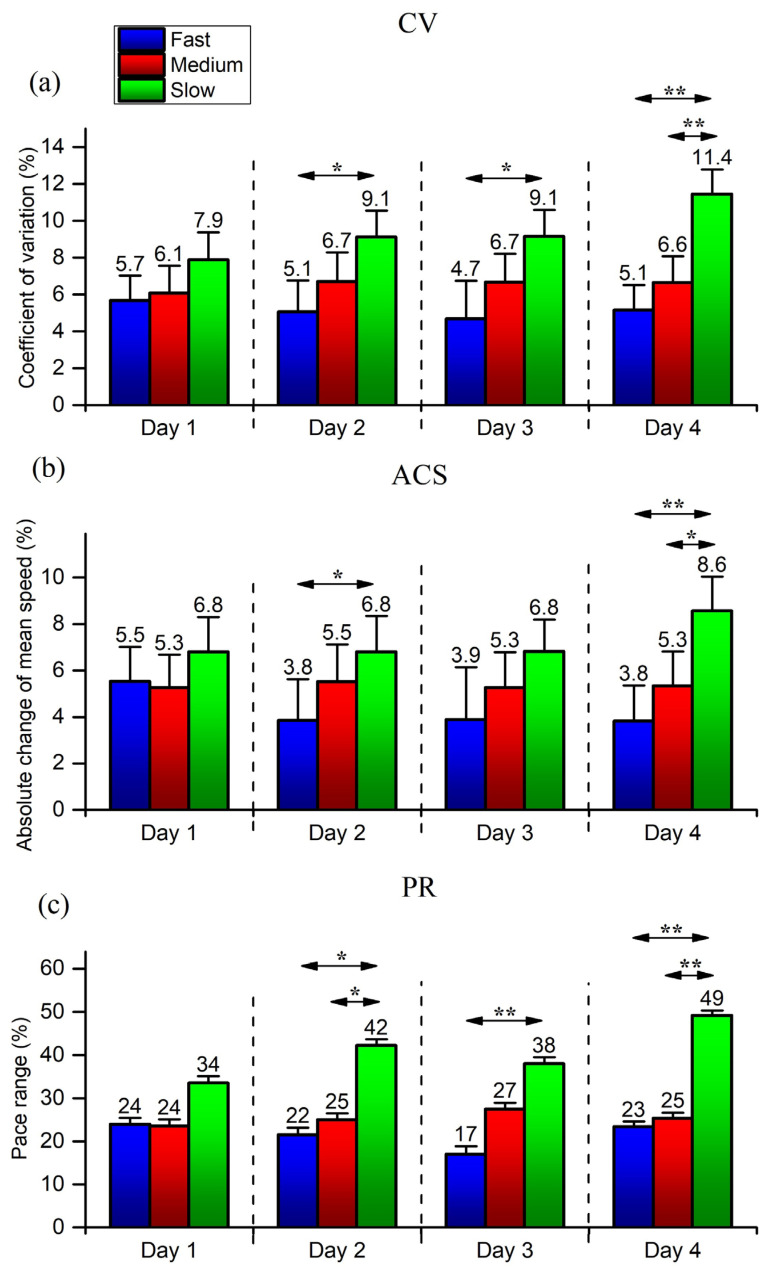
Differences between 3 performance groups for CV (**a**), ACS (**b**) and PR (**c**) for all four days. CV—Coefficient of variation; ACS—Absolute change in mean speed; PR—Pace range; * *p* < 0.05; ** *p* < 0.01.

Furthermore, CV, ACS, and PR prove sensitive enough (Figure 4) to detect differences between the three performance groups. In particular, these differences can be observed in the last three days when the Slow group is compared to the Medium and Fast groups.

## 4. Discussion

This study aimed to assess the long-distance running variability and the reliability, validity, and sensitivity of commonly used pacing variables in four marathons over four days. The first hypothesis that multi-stage marathon running would show low pacing variability in both individual and mean results was confirmed. Furthermore, several pacing variables (CV, ACS, and PR) would show more reliability, validity, and sensitivity to be routinely used in future studies, thus confirming the second hypothesis. Finally, the CS, CSF, MRS, and 32-10 pacing variables showed low reliability and validity indices and should be used with caution in future studies.

### 4.1. Running Variability

Previous studies mostly detected low day-to-day pacing variability in swimming, cycling, and middle-distance running [28,30,32]. Although this study involved much longer distances and significantly more split times (33 per day, 132 in total), day-to-day pacing in the multi-stage long-distance running event is also relatively constant and specific for each runner (see Figure 2).

Pacing regulation in the long-distance running seems to occur with both feedback and feedforward control, with continuous integration of these two components [9]. As a result, the pacing might be perceived as a self-regulatory learning skill that needs to be developed over the years and cannot change easily [47]. Specifically, all runners in our study had the same pacing profile in all 4 days or 3 out of 4 days. Although their pacing profiles were mainly positive, no significant decrease in running speed was observed (Table 1). It appears that repeating the pacing strategy from day to day was the participants’ choice or something they had learned over time, thus emphasizing the importance of the cognitive resources required in regulating exercise intensity [48]. For example, athletes with an intellectual impairment appeared to have difficulties efficiently self-regulating their pace [49]. Therefore, we can argue that selecting the most appropriate pacing strategy was based on all perceived action possibilities. It is a skill that can be learned and developed over the years [5]. It should be noted that in this particular case, participants were mainly experienced, master runners (mean age 55.5 years ± 9.5 years), which might have contributed to the more stable running variability.

These findings (i.e., low day-to-day running variability) provided an ideal opportunity to test the reliability and, consecutively, validity and sensitivity of the commonly used pacing variables (expressed as some form of speed change).

### 4.2. Reliability and Validity of the Pacing Variables

The pacing variables, CS, CFS, MRS, and 32-10, often utilized in pacing studies [3,17,20,41], showed relatively low reliability and validity indices. Specifically, in CS, both negative and positive splits are compared together, which could indicate falsely low variability while the variability is actually high. Numerous studies have used this variable, and many did not show how they addressed this issue [20,38]. CSF is often used in studies examining elite runners [3]; however, some studies used this variable to assess recreational runners’ pacing [39]. Recreational runners usually start the race more rapidly [18] since they are not experienced in controlling their pacing behavior. As a result, variables derived from the speed change of the first split time (such as CSF) can be unreliable. The MRS pacing variable is practical to assess pacing in recreational 10 km races where usually only one split time is provided (e.g., at 5 km) [7]. However, some studies utilized this variable even when multiple split times were available [42]. Finally, March et al. [17] provided a reasonable and physiologically excellent explanation for the 32-10 pacing variable. However, like in MRS, it appears that using only two split times to obtain the pacing variable decreases its reliability and validity. Furthermore, many races do not provide specific split times, which are needed to calculate the 32-10 variable.

On the other hand, both the CV and ACS consider mean speed in the calculation while requiring more than two split times. This might contribute to better reliability (Table 1). These results are in agreement with the previous studies in middle-distance running (CV 1.2–11.5% [32]), kayaking (ICC 0.644–0.996 [26]), cycling (ICC > 0.870 [31]), and swimming (CV < 5% [27]). Although the CV is regarded as the golden standard in assessing variability [45], the CV as a pacing variable does not provide enough practical applications [23]. Contrary to ACS, the CV cannot be used for evaluating pacing for separate race segments (e.g., 5 km, 10 km). Furthermore, the ACS uses only absolute values, thus accurately showing a total change in pace. Since even pacing strategy appears to be the best strategy, particularly for recreational long-distance runners [8,10,11], ACS can be routinely used in the pacing analysis of mass participation events.

Besides CV and ACS, PR was also a reliable and valid pacing variable. Nevertheless, the downside of the PR variable can be observed when few or too many split times are provided since one very fast or one very slow split can add too much variability. This might occur more often in inexperienced runners who do not have the same physical capacity as elite runners to maintain a consistent pace [37]. Also, PR does not include an end spurt because premature fatigue reduces as the endpoint approaches. As a result, the reserve that runners maintain for most of the race to reduce the hazard of catastrophic collapse can be unleashed [12], thus increasing pacing variability.

### 4.3. Sensitivity of the Pacing Variables

Since the CS, CSF, MRS, and 32-10 variables showed poor reliability and validity indices, they were not considered for further sensitivity analysis.

Previous studies showed that pacing is more variable in slower runners and even in faster runners [20,42]. Fast runners maintain greater velocities over the entire distance to achieve a faster finishing time [50], thus reducing running variability. In contrast, a large running variability would mean the runner is undergoing extensive changes in velocity, which would seem detrimental to elite marathon performance and is often observed in recreational runners [37]. Therefore, we assumed that sensitive pacing variables might identify this variability.

Indeed, CV, ACS, and PR all showed a moderate to high, negative correlation with the running speed (i.e., more variability equals lower running speed) while detecting differences between three performance groups (mainly between the slow and the other two faster groups; Figure 3). There were no significant differences between the fast and medium groups, which the low number of participants might explain. Pacing in long-distance running is also influenced by age and gender [18,40]. However, this study did not compare men and women and did not include young and old runners (i.e., younger than 30 and older than 70), whose pacing is more variable than that of the middle-aged runners [40].

### 4.4. Limitations

Several limitations must be acknowledged for this study. Firstly, runners had to adapt their pace to daily running and a cumulative race. However, it appears that pacing strategy is a skill that can be taught [5,47], and it was not significantly affected by day-to-day running. Secondly, although participants in this study maintained running speed throughout the days, potential undetected fatigue in multi-stage events might have influenced pacing. Thirdly, no information was recorded regarding the participant’s anthropometric data or training history. Finally, sensitivity analysis was only performed with regard to the race speed and performance groups. Nevertheless, these participants ran in ideal conditions according to their abilities in “ecologically valid” conditions.

## 5. Conclusions

In conclusion, multi-stage marathon running showed low variability in individual and group results. The pacing variables CV, ACS, and PR proved reliable, valid, and sensitive enough to be routinely used in future studies exploring pacing in long-distance running.

### Practical Applications

Researchers and practitioners (i.e., running coaches and sports scientists) who aim to explore pacing in long-distance running in recreational runners should utilize ACS or CV variables in their analysis. Furthermore, if standardized race segments exist (e.g., 5 km, 10 km), ACS should be used instead of CV, while PR can be a good option if a race includes more elite runners. Specifically, by implementing these recommendations, researchers might (re)discover some new phenomenon regarding pacing differences in different populations or conditions. On the other hand, post-hoc pacing analysis can help runners better manage their energy to avoid burnout and injuries, and consecutively achieve their running goals.

Future studies could use similar multi-stage events to explore the minimum number of split times (race segments) needed to obtain reliable and valid pacing variables. In addition, future studies might try confirming these results using different multi-stage event’s data as well as expanding sensitivity analysis to age and gender differences.

## Figures and Tables

**Table 1:** Indices of reliability of mean running speed and the commonly used pacing variables.

Variable	Day 1	Day 2	Day 3	Day 4	CV%	SEM	ICC (95% CI)	F	*p*
MS	2.72 ± 0.32	2.74 ± 0.36	2.73 ± 0.36	2.69 ± 0.38	4.10	0.11	0.920 (0.862, 0.958)	0.731	0.538
CV	6.50 ± 1.47	6.85 ± 1.64	6.68 ± 1.76	7.44 ± 1.58	23.22	1.26	0.785 (0.650, 0.884)	1.244	0.302
CS	5.58 ± 2.30	4.27 ± 2.47	5.17 ± 2.76	3.52 ± 3.17	77.09	2.16	0.404 (0.190, 0.625)	1.110	0.353
CSF	9.69 ± 1.61	7.49 ± 1.49	9.02 ± 1.99	7.46 ± 1.51	43.64	1.55	0.267 (0.06, 0.509)	2.109	0.130
ACS	5.84 ± 1.47	5.32 ± 1.69	5.25 ± 1.76	5.69 ± 1.67	24.42	1.28	0.781 (0.645, 0.882)	0.775	0.513
PR	26.78 ± 1.56	28.70 ± 1.65	26.62 ± 1.77	31.20 ± 1.51	26.10	1.30	0.732 (0.576, 0.853)	1.721	0.173
MRS	4.05 ± 3.24	4.21 ± 4.10	6.32 ± 2.45	4.55 ± 2.01	93.84	2.56	0.258 (0.052, 0.501)	0.864	0.465
32-10	5.45 ± 2.59	3.96 ± 2.94	4.87 ± 2.51	3.24 ± 3.57	82.54	2.28	0.418 (0.205, 0.637)	1.112	0.342

Abbreviations: SEM—Standard error of measurement; CV%—Coefficient of variation; ICC—Intraclass correlation coefficient; CI—Confidence Interval; F—ANOVA; *p*—Level of statistical significance; MS—Mean speed; CV—Coefficient of variation; CS—Change in mean speed; CSF—Change in the first lap speed; ACS—Absolute change in mean speed; PR—Pace range; MRS—Mid-race split; 32-10—First 32-10 split.

**Table 2:** The pacing variables’ concurrent validity compared to the CV for four running days.

Variables (Day 1)	CV (Day 1)		Variables (Day 2)	CV (Day 2)		Variables (Day 3)	CV (Day 3)		Variables (Day 4)	CV (Day 4)	
	r	*p*		r	*p*		r	*p*		r	*p*
CS	0.416	0.068	CS	0.093	0.695	CS	0.010	0.968	CS	0.323	0.165
CSF	0.317	0.173	CSF	0.664 **	0.001	CSF	0.712 **	<0.001	CSF	0.828 **	<0.001
ACS	0.926 **	<0.001	ACS	0.982 **	<0.001	ACS	0.980 **	<0.001	ACS	0.977 **	<0.001
PR	0.972 **	<0.001	PR	0.924 **	<0.001	PR	0.969 **	<0.001	PR	0.908 **	<0.001
MRS	−0.193	0.415	MRS	0.389	0.090	MRS	0.418	0.067	MRS	0.657 **	0.002
32-10	0.396	0.084	32-10	0.090	0.706	32-10	0.039	0.870	32-10	0.314	0.178

Abbreviation: CV—Coefficient of variation; CS—Change in mean speed; CSF—Change in the first lap speed; ACS—Absolute change in mean speed; PR—Pace range; MRS—Mid-race split; 32-10—First 32-10 split; r—Correlation coefficient; *p*—Level of statistical significance; ** *p* < 0.01.

**Table 3:** Sensitivity of the pacing variables—correlation of the CV, ACS, and PR compared to the MS.

Variables (Day 1).	Mean Speed (Day 1)		Variables (Day 2)	Mean Speed (Day 2)		Variables (Day 3)	Mean Speed (Day 3)		Variables (Day 4)	Mean Speed (Day 4)	
	r	*p*		r	*p*		r	*p*		r	*p*
CV	−0.480 *	0.032	CV	−0.531 *	0.016	CV	−0.560 *	0.010	CV	−0.709 **	<0.001
ACS	−0.409	0.073	ACS	−0.461 *	0.041	ACS	−0.479 *	0.033	ACS	−0.669 **	0.001
PR	−0.520 *	0.019	PR	−0.632 **	0.003	PR	−0.623 **	0.003	PR	−0.670 **	0.001

Abbreviation: CV—Coefficient of variation; ACS—Absolute change in mean speed; PR—Pace range; r—Correlation coefficient; *p*—Level of statistical significance; * *p* < 0.05; ** *p* < 0.01.

## Data Availability

The datasets analyzed during the current research are available from https://www.sporkrono.fr/2021-resultats-bretzel-ultra-triathlon/ and https://kachelmannwetter.com/ (accessed on 10 October 2021).
